# State Board of Health

**Published:** 1876-03

**Authors:** 


### Editorial.

State Board of Health.—We desire again to call the attention of the medical profession to the great work which may be accomplished by the State Board of Health, so successfully inaugurated, provided it can have the general and cordial support of the medical men of the State. While the law has certainly, in our judgment, been seriously impaired in its force and efficiency by abolishing, as has been done by the following amendment passed at the last session of the Legislature, the compulsory feature or penalty for non-compliance with its provisions. Under any circumstances its full success was dependent upon the appreciation of its importance by those upon whom the duty of making reports devolved. This, to a very large extent, of course, rested upon medical men, and we do most earnestly urge upon the profession of this State not to allow this most conspicuous feature of our advancing civilization to be blotted out by a failure upon their part to uphold the arms of the State Board of Health in this important part of the organization, from which so much of priceless value may be realized with proper co-operation upon the part of the physicians of the State, and without which aid the Board will be literally powerless to accomplish any valuable result in connection with registration. There is, however, one redeeming feature in the amendment alluded to, in the provision for the establishment of County Boards of Health, composed of two physicians and the Ordinary, from which we can but hope such valuable aid may be obtained in carrying out the objects of the Board as to largely compensate for the loss of the compulsory feature. We hope that immediate steps will be taken in every county in the State to have these Boards organized in accordance with the provisions of the following amendment, and the most efficient medical men, and those only who are determined to carry out the law, will be appointed :

AN ACT

"To amend an act, entitled an act to create a State Board of Health for the protection of life and health, and to prevent the spread of diseases in the State of Georgia, and for other purposes, approved February 25th, 1875, and for other purposes.

"Section 1. *Be it enacted by the General Assembly of the State of Georgia*, That so much of the 11th section of the act entitled an act to create a State Board of Health, for the protection of life and health, and to prevent the spread of diseases in the State of Georgia, and for other purposes, approved February 25th, 1875, as imposes a penalty of ten dollars upon physicians, be and the same is hereby repealed.

"SEC. 2. *Be it further enacted by the authority aforesaid,* That so much of the 12th section of said act as imposes the penalty of ten dollars on parents is also hereby repealed.

"SEC. 3. *Be it further enacted by the authority aforesaid,* That for making the record of births and deaths, as now required by law, the Ordinaries of the several counties of this State shall recieve five cents for each person so registered.

"SEC. 4. *Be it further enacted by the authority aforesaid,* That County Boards of Health are hereby created, to consist of the Ordinary, who shall be *ex officio* a member of said board, and two practicing physicians, to be chosen by the Commissioners of Roads and Revenue, in those counties where such commissioners are allowed by law, and in other counties by the grand jury of the first term of the court held after the passage of this act, and the term of office of the members of said county boards shall be the same with Ordinary.

"SEC. 5. *Be it further enacted by the authority aforesaid,* That the Ordinary of each county shall be *ex-officio* secretary of the county board for his county, and said board shall correspond with, and report statistics to, the State Board of Health through its secretary, giving special attention to gathering statistics in accordance with the provisions of the act of which this is amendatory.

"SEC. 6. *Be it further enacted by the authority aforesaid,* That said county boards shall have supervision of the sanitary condition of their counties respectively, subject to the directions of the State Board of Health.

"SEC. 7. Repeals conflicting laws."

